# Washing out the Stomach

**Published:** 1874-11

**Authors:** 


Washing out the Stomach.—Dr. C. Ewald, of Berlin, describes a method of washing out the stomach, which, on account of its great simplicity, seems likely to make the topical treatment of diseases of the stomach, especially in cases of poisoning, much more common: "A piece of ordinary india-rubber tubing, such as is used for gas-lamps, about six feet long, is used; one end is rounded with scissors, and, if necessary, two holes are cut at a short distance from the end. This tube possesses quite sufficient rigidity to be passed without difficulty into the stomach. To the outer end a funnel is fitted, into which can be poured either water or a solution of soda, etc., according to circumstances. If the contents of the stomach are to be removed, the outer end of the tube must be sunk to the level of the pubes, or even lower; then the patient must make a short but forcible contraction of the abdominal walls. By this means the tube is filled to its highest point with the fluid contents of the stomach, and becomes a siphon; the liquid continuing to flow until there is no more, or until the tube is stopped up. This last seldom occurs, if the tube be of a moderate calibre. Should it, however, happen, or should the abdominal pressure be insufficient to fill the tube in the first instance, or the patient be insensible, or any similar difficulty arise, it can, in general, be readily overcome by fitting a common clyster-syringe to the end of the tube, one stroke of the piston of which is generally sufficient to remove the difficulty."—*Journal of Chemistry.*

